# Evolution and Selection in Yeast Promoters: Analyzing the Combined Effect of Diverse Transcription Factor Binding Sites

**DOI:** 10.1371/journal.pcbi.0040007

**Published:** 2008-01-11

**Authors:** Daniela Raijman, Ron Shamir, Amos Tanay

**Affiliations:** 1 School of Computer Science, Tel Aviv University, Ramat Aviv, Israel; 2 Department of Computer Science and Applied Mathematics, The Weizmann Institute, Rehovot, Israel; Max Planck Institute for Molecular Genetics, Germany

## Abstract

In comparative genomics one analyzes jointly evolutionarily related species in order to identify conserved and diverged sequences and to infer their function. While such studies enabled the detection of conserved sequences in large genomes, the evolutionary dynamics of regulatory regions as a whole remain poorly understood. Here we present a probabilistic model for the evolution of promoter regions in yeast, combining the effects of regulatory interactions of many different transcription factors. The model expresses explicitly the selection forces acting on transcription factor binding sites in the context of a dynamic evolutionary process. We develop algorithms to compute likelihood and to learn de novo collections of transcription factor binding motifs and their selection parameters from alignments. Using the new techniques, we examine the evolutionary dynamics in *Saccharomyces* species promoters. Analyses of an evolutionary model constructed using all known transcription factor binding motifs and of a model learned from the data automatically reveal relatively weak selection on most binding sites. Moreover, according to our estimates, strong binding sites are constraining only a fraction of the yeast promoter sequence that is under selection. Our study demonstrates how complex evolutionary dynamics in noncoding regions emerges from formalization of the evolutionary consequences of known regulatory mechanisms.

## Author Summary

Cells use sophisticated regulation to transform static genomic information into flexible function. We are still far from understanding how such regulation evolves. Short DNA sequences that physically bind transcription factors in promoter areas near target genes play an important role in gene regulation and are directly subject to mutation and selection. In this work, we develop a methodology for studying the evolution of promoter sequences under the effect of multiple regulatory interactions. We present a model that describes the evolutionary process at each genomic locus, taking into account a random flux of mutations that occur in it and the effects of transcription factor binding sites gain or loss. Our model accounts for dependencies (or epistasis) between adjacent loci that contribute to the same regulatory interactions: mutation in one such locus immediately changes the effect of mutations in the other. Using our model, we characterize the evolution of promoters in yeast, showing that many regulatory interactions that were discovered experimentally or computationally are evolutionarily unstable. The dynamic nature of transcriptional interactions may be explained if the regulatory phenotype is achieved through multiple interactions at different levels of specificity, and if only relatively few of these interactions are essential for themselves.

## Introduction

Genomic regulatory regions harbor complex control schemes that collectively allow the genome to operate in a flexible and dynamic fashion. Such control schemes are encoded into the DNA sequence in a way that is not yet fully understood. Important elements of such regulatory code are short DNA sequences that are bound by transcription factors (TFs). TFs bind regulatory DNA specifically, by recognizing short motifs, and contribute to the assembly of complex switches that govern the transcription of a gene, given various environmental or internal signals. Much of the current understanding of the way in which DNA determines the regulatory program of a gene is based on identification of TF binding sites (TFBSs) and their association with TFs of known function.

Despite remarkable progress in functional genomics technologies, and in the ability to experimentally profile TF–DNA interactions on a genomic scale [1,2], the understanding of function in regulatory regions remains a major challenge. At the same time, the complete sequencing of evolutionarily close genomes has made the detailed comparative study of regulatory regions possible. Consequently, comparative genomics has emerged as one of the central ways by which regulatory signals are computationally detected and studied. All comparative methods assume (explicitly or implicitly) an evolutionary model that distinguishes neutral sequences from functional ones. Most commonly [3–7], comparative studies focus on conservation, classifying sequences to be functional or nonfunctional by assuming that evolution in functional loci is slower. In yeast, many conserved loci were shown to correspond to TFBSs, allowing detection of novel sites that were not identifiable using single species methods.

As more species are sequenced, a desirable challenge is to extend the simple conservation-based studies by adding more structure to the function–evolution relationship in regulatory regions. In coding regions, our understanding of the genetic code makes sophisticated evolutionary predictions possible, e.g., by identifying cases of positive selection [8], correlated residues [9], and more. It is hoped that by acquiring better, more detailed understanding of the function encoded by regulatory loci, one can greatly extend the utility of comparative studies in a similar way.

In this study, building on simple assumptions of the mechanisms of transcriptional regulation, we formalize an evolutionary model combining a neutral mutational process with selection on multiple heterogeneous TFBSs. We develop techniques for computing the likelihood of such a model given pairwise alignments and for learning maximum-likelihood model parameters. Using the new techniques, we can express a substantial part of the current functional knowledge on gene regulation in evolutionary terms and evaluate observed patterns of divergence and conservation based on this model. Applying our method to promoter sequences of *Saccharomyces* yeast species, we validate our approach and exemplify its use. Specifically, we discuss how the selection on binding sites of different TFs vary in intensity, and how some families of similar TFBSs are in fact divided into subgroups that are separated by selection. We compute the fraction of promoter sequence that is under selection due to characterized TFBSs and show that strong TFBSs constitute only a small fraction of the promoter sequences in yeast. The gap between selection due to strong TFBSs and global estimates of the selection on yeast promoters can be used to estimate the relative roles of classical binding sites and of other effects (low affinity transcriptional interactions, and possibly other factors, e.g., chromatin organization) in driving functional transcriptional networks.

## Results

### Probabilistic Modeling of Promoter Evolution

**Figure 1:**
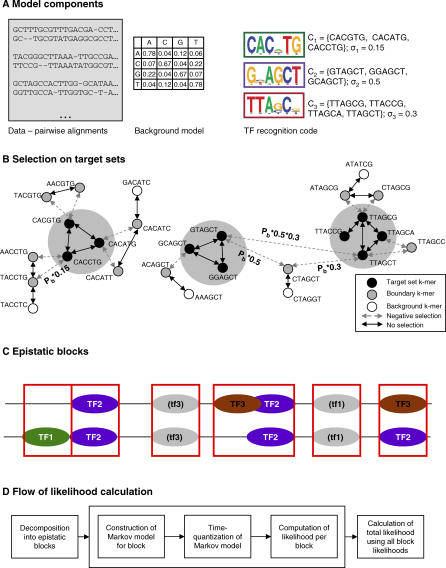
An Integrated Model for Evolution under the Effect of Multiple TFs (A) A TF recognition code. The TF recognition code model is defined by a set of target k-mer sets identified by specific TFs, and selection factors that quantify the intensity of selection on substitutions affecting them. In this example, the model consists of three target k-mer sets for three TFs. The background substitution probabilities are also part of the model. (B) Selection on k-mers. Substitutions between k-mers that belong to a target k-mer set (large grey circles) and k-mers that do not belong to that target k-mer set are under negative selection. The probability for that substitution is derived by multiplying the background probability by the appropriate selection factor(s). (C) Epistatic blocks. To compute the likelihood of a model given alignments, we first decompose the sequence into epistatic blocks that evolve almost independently of each other. Nontrivial epistatic blocks (marked by rectangles) include overlapping TFBSs or k-mers that are one substitution away from a target k-mer set (boundary k-mers). Ovals marked TFX represent a TFBS for TF X. Ovals marked (tfX) represent boundary k-mers for TF X. Loci that are not related to any TF form trivial epistatic blocks of size 1, and are not shown. (D) Scheme for computing likelihood. We approach the complex task of computing likelihood in our model by decomposing the sequence to epistatic blocks and computing likelihood inside each of them.

We developed an integrated model for the evolution of promoters under the influence of heterogeneous TFBSs (Methods). Briefly, the *TF recognition code* is a collection of distinct DNA motifs, where each motif (corresponding to a TF) is represented by a set of nucleotide k-mers. We assume that each set (termed *target k-mer set*) contains all k-mers recognized by the TF (see Figure 1A for an illustration), and any appearance of a k-mer from the target k-mer set is declared to be a binding site. All k-mers in the same target k-mer set are of the same length, but are otherwise unconstrained. In practice, target k-mer sets are usually variations over a consensus sequence. Our model represents a simplification of a much more complex biological reality, by assuming that binding at each locus is completely determined by the existence of a motif, and is either perfect or nonexisting (therefore ignoring differences in binding affinity between k-mers of the same target k-mer set). These simplifications allow us to develop a model for which computation is practical, but should be carefully evaluated and eventually relaxed in future revisions of the model.

To model the evolution of a promoter region, we assume that sequences are evolving neutrally, except for loci affected by selection on TFBSs. Each target k-mer set (and therefore each TF) is associated with a *selection factor* 0 ≤ σ ≤ 1, which represents the relative fixation probability of a mutation introducing or eliminating a binding site (note that the selection factor is not equivalent to the classical selection coefficient). Smaller σ values represent stronger selective pressure on loci bearing k-mers that belong to a given target set. Our model assumes that each appearance or loss of a TFBS is selected against. The replacement of a k-mer from a target set by another k-mer from the same target set is not selected against, since according to our model all k-mers in the same target set are equivalent. This simple functional model allows us, once equipped with a TF recognition code, to write down a Markov model representing the evolution of an entire promoter sequence.

The evolutionary forces outlined in our model affect the rate of mutation at a particular single base if it is in the context of a TFBS. The evolution of one base can therefore depend on several adjacent bases, and the model formalizes this type of *epistasis* using the simple assumptions of TFBSs described above. Although the epistasis considered by the model is simple and spatially limited (including only binding site k-mers), exact computation of the likelihood of a TF recognition code given a multiple or even pairwise alignment is very difficult and involves exponentiation of a 4^l^ by 4^l^ matrix, l being the sequence length. We developed algorithms for approximate calculation of the likelihood of a model, which provide us with a method for evaluating to what extent our model agrees with the patterns of divergence in the alignment. We score models by comparing their likelihood to that of a null model representing neutral substation rates on independent loci, deriving a log-likelihood ratio (LLR) score. Using these tools, we can search for maximum likelihood selection factors for a given recognition code, e.g., based on available experimental information. We can also learn a recognition code de novo directly from alignments and study the collective evolution of a group of TFBSs in an unbiased fashion (Methods).

We note that our framework was not designed as an attempt to develop another TFBS motif finding algorithm, a problem that is already treated extensively in the literature [10].

We focused on the evolutionary dynamics of *Saccharomyces* gene regulatory regions. The yeast system has the advantage of many well-documented TFBS motifs and clearly identifiable promoters, and was used before in many studies of transcriptional regulation and its evolution [4,6,11]. We extracted pairwise alignments from multiple alignments of *Saccharomyces sensu stricto* species (Methods). For example, the resulting alignments for *S. cerevisiae–S. mikatae* consisted of more than 900,000 aligned bases from the upstream regions of 3,503 genes, with 74.2% identity. Alignments of *S. cerevisiae–S. bayanus* and of *S. cerevisiae–S. paradoxus* were also used (Table S1).

### Literature-Based TFBSs

We started by constructing an evolutionary model from known TF binding models. We used the compendium of TFBSs composed by MacIsaac et al. based on extensive ChIP-on-chip data and literature review [12]. Out of 124 consensus sequences reported by the authors (in IUPAC format), we chose those 94 that translated to target k-mer sets containing at most 512 k-mers each, and had at least five matches in the aligned *S. cerevisiae–S. mikatae* promoters. We constructed a model starting from an empty one, and incrementally attempting to add each of the 94 target k-mer sets (in an arbitrary order). For each candidate target k-mer set in turn, we tentatively added it to the model and inferred an optimal selection factor for it. We then tested whether the expanded model with the added target k-mer set had a selection factor smaller than 1 and a higher model likelihood (note that a target k-mer set with a selection factor equal to 1 would result in a log-likelihood ratio of 0). If this was the case, the target k-mer set was accepted to the model. Otherwise it was rejected and not kept in the model. In total, we accepted 74 target k-mer sets (79%). Similar results were obtained for the other two yeast species.

We next studied possible factors that contribute to acceptance or rejection of literature target k-mer sets in our model. According to our model, every appearance of a motif is considered to be functional and under selection. In reality, not all appearances are necessarily functional, and some may be functionally different than others. We examined the correlation between the number of appearances of k-mers from a target k-mer set in the data and the model acceptance rate. While 87% of the target k-mer sets with 0–149 hits were accepted, only 77% of target k-mer sets with 150–499 hits were accepted, and only 47% of the target k-mer sets with more than 500 hits were accepted. These results suggest that the specificity of some of the literature target k-mer sets may be too low to allow acceptance by our rather stringent model.

To try to control for motif specificity in a systematic way, we next examined, for each TF, a model constructed using a limited dataset, containing only pairwise alignments of promoters that were found to be bound by that TF in ChIP-on-chip experiments (using a *p* < 0.005 cutoff) [2,13]. Since the set of ChIP-bound promoters is different for each TF, we could not construct a complete model in this case, but simply computed the LLR for each TF in a model containing a single k-mer target set. We call the resulting model the *ChIP model*. Out of 62 TFs with at least five hits in the ChIP bound promoters, 52 target k-mer sets had positive LLR at σ < 1 (84%), of which six were not accepted in the original model (Spt2, Ndd1, Swi5, Bas1, Hap2 and Met31). All of the target k-mer sets that were accepted in the original model but not in the ChIP model (27 in total) had fewer than five hits in the ChIP data and therefore were not considered. In summary, although our model assumptions are simplistic, they are enough to roughly approximate the behavior of a large fraction of the known binding sites in the yeast genome. The cases of known TFBSs whose evolution is not well captured by the model are not resolved by restricting the analysis to experimentally verified TF targets, suggesting that the simple association between motifs and function does not hold for them.

### Learning a Nonredundant TF Recognition Code De Novo

**Figure 2:**
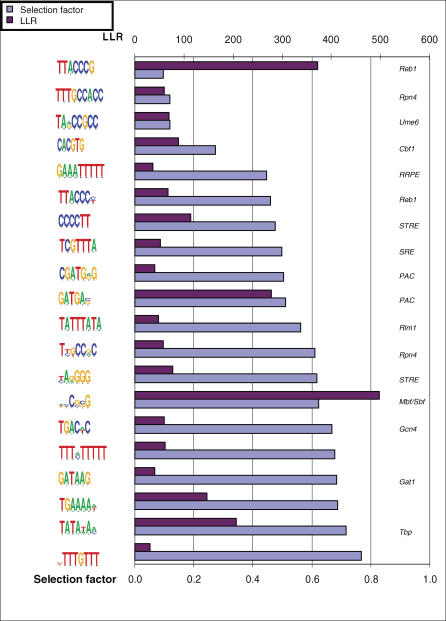
De Novo Model Shown are the LLR and selection factor values for the 20 target k-mer sets with the highest LLR values as discovered in the de novo model. The full model is presented in Table S2. For each target k-mer set, we present its motif logo, and, when the set matches a published binding site motif, its most likely TF. Target k-mer sets are sorted according to selection factor, indicating many of the known TFs as having relatively high factor and weak selection. Also shown is the LLR of each target k-mer set, which is affected by both the number of motif appearances and their divergence properties.

We next applied our model learning algorithm to construct a TF recognition code model de novo. By constructing a de novo model we were not hoping to discover new TFBS motifs, but rather to study the evolutionary dynamics of the yeast promoters given the selection on an unbiased set of putative TFBSs. The model was constructed automatically, considering gapless k-mers of width 6–12 as candidate target k-mer seeds (Methods). The learning algorithm produced a model containing 62 target k-mer sets when executed on the S. cerevisiae–S. mikatae alignments (see Figure 2 and Table S2). The de novo target k-mer sets matched 45 distinct known motifs (Methods). We note that most of the target k-mer sets that we learned are relatively specific, with no or limited redundancy, and that we preferred a larger model over a more stringent one, to allow global properties of the model to be explored.

In our modeling framework, it can be assumed that each inferred target k-mer set represents a distinct function and that no two target k-mer sets that represent the same function coexist in the model. The reason for this is that substitutions between k-mers in two equivalent target k-mer sets of the same TF would be predicted by the model to be selected against (multiplying the probability of neutral substitution by the selection factors of each of the target k-mer sets), while, in fact, substitution between redundant target k-mer sets must behave neutrally. This discrepancy should result in lower likelihood for a model that includes two redundant target k-mer sets instead of just their union. Looking at the results, we indeed see only a few cases of seemingly redundant target k-mer sets, each of which can be biologically rationalized as described below.

**Figure 3:**
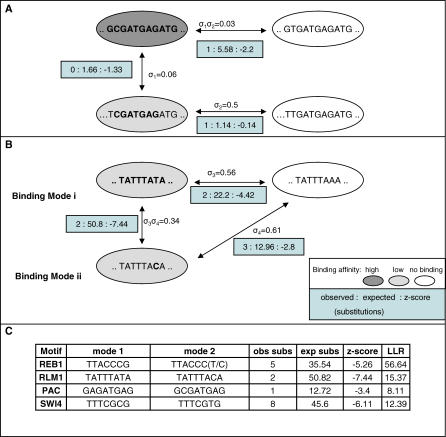
Overlapping and Bimodal Target k-mer Sets (A,B) We demonstrate the effect of similar motifs in two types of redundancies, using examples from the de novo model. In one type of redundancy (A), two motifs are overlapping (here GCGATGAGATG and CGATGAG). This organization corresponds to one effective motif with two levels of affinity. The strong affinity binding sites appears as a hit for both motifs, and therefore the selection on it is the combined selection of both motifs. The weak affinity binding sites appears as a hit for just one motif (CGATGAG). A different type of redundancy (B) associates two similar motifs that differ in one base (here TATTTATA and TATTTACA), suggesting selection is preserving the separation between two variants of the same motif. We quantify the intensity of separation by comparing the likelihoods of models with merged and separated target k-mer sets, or by directly assessing the rate of substitutions between motifs in the two target k-mer set variants (Text S1). (C) Inferred bimodal motifs. Shown are cases of similar but separated target k-mer sets in the de novo model, indicating the number of observed and expected substitutions between them, a z-score for these numbers (see Methods), and the LLR of the merged and separated models.

In the first type of model redundancy, one target k-mer set contains substrings of another (e.g., GCGATGAGATG and CGATGAG in the PAC motif). This can be accounted for by the inaccuracy of the discrete binding assumption. If one target k-mer set represents strong (more specific) binding sites and the other represents weaker sites, then having two target k-mer sets with different selection factors improves likelihood. In this case, according to our model the selection on a locus with the more specific version is calculated as if it were part of binding sites for both target k-mer sets (implying a de facto stronger selection on it). In contrast, the selection on a locus with the less-specific version would be affected only by the selection factor of one target k-mer set. This is exemplified in Figure 3A.

In the second type of model redundancy, two k-mers from distinct target k-mer sets differ in one position (e.g., TTACCCG and TTACCCT in the Reb1 motif, TATTTATA and TATTTACA in the Rlm1 motif). In this case, the likelihood of a model in which the two target k-mer sets are combined into one is lower than the likelihood of the redundant model. This suggests that the separation between the two target k-mer sets is selected for, possibly since BSs from each set are functioning differently (Figure 3B). Examples for such separation were argued for heuristically and demonstrated experimentally before [11,14], but now we are equipped with the computational means to quantify such selection.

As shown in Figure 3C, substitutions between target k-mer sets that are seemingly redundant can be directly shown to occur at a lower rate than expected using Z-score statistics (Methods), as well as using the LLR of the redundant and combined models. The cases we observed include the previously discussed Reb1 motifs [11,15,16] and separation among variants of the still cryptic PAC motif. PAC targets are highly enriched in stress response genes [17], but the mechanisms of PAC based regulation are not well characterized. We discovered two separated PAC-like families (GCGATGAG and GAGATGAG) that are significantly separated from each other. Interestingly, both variants of the PAC model tend to co-occur in the same promoters with the RRPE motifs (co-occurrence Z scores of 15.5 and 15.6), suggesting that they share a common mechanism rather than representing two distinct factors.

### TFBS Selection Factors

**Figure 4:**
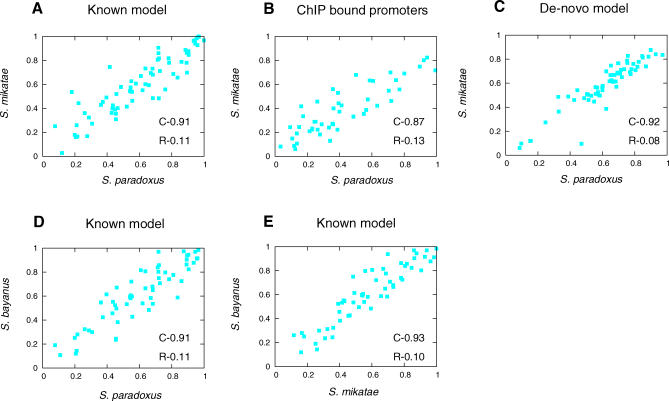
Selection Factors Shown are inferred selection factors, compared for pairs of similar target k-mer sets on alignment of S. cerevisiae promoters with different yeast species. *C*, the correlation between the σ values in the two models. *R*, the root mean square difference value. The high correlation among the inferred σ values indicates that our model successfully decomposes the background mutation rate (which is different for each species) and the selection on each TFBS (which is quite stable as seen here). (A,D,E) Comparison of σ values in the literature-based model for each pair of species. (B) The literature-based model using ChIP-bound promoters only on *cerevisiae-paradoxus* alignments compared to the same model on *cerevisiae-mikatae* alignments. (C) The de novo model on *cerevisiae-paradoxus* alignments compared to the same model on *cerevisiae-mikatae* alignments.

An important characteristic of our model is the separation between background substitution rates and the selection factor on target k-mer sets (σ values). Since we analyzed separately pairwise alignments of three different species with S. cerevisiae, and since these species differ significantly in their divergence times from S. cerevisiae, we can compare the σ values of the same TFBS obtained in each pair of species. We can attribute differences in such σ values to changes in selective pressure or to other TF-specific effects (like divergence of the TF itself), rather than to different divergence times between species pairs or other background effects. According to the results (Figure 4) the σ values of the same TFBS are similar across the different species pairs (Spearman correlation values ranging around 0.9), even though some species pairs are four times as distant evolutionarily [4–7,18], suggesting that these values represent a quantity that is by and large independent of background divergence.

We observed significant variability in the inferred selection factor of known TF motifs in the literature-based target k-mer sets. Many of the well-known TFs with low degeneracy target k-mer sets (<8 k-mers) had small σ values, suggesting specific binding and tight selection. Some examples are Reb1 (0.18), Rpn4 (0.16), Ume6 (0.03), and Leu3 (0.12). However, for other well-known motifs we derived much higher σ values. These include the CACGTG motifs (Cbf1, Pho4, Tye7, and Met28) (0.4–0.91), Mbp1 (0.46), Swi6 (0.54), and Msn2/4 (0.54). Interestingly, we inferred high σ values (>0.35) for these TFs in the ChIP-restricted model, too (see Table S3 for σ values computed for different ChIP thresholds). This suggests that the mild selection factors for these TFBSs are not primarily a side effect of false positives, since it is widely assumed that motifs in promoters that are also ChIP targets are very likely to be bound in vivo.

One possible explanation for the reduced selection on some of the target k-mer sets may be that k-mers from these sets tend to appear in multiple copies in each of the promoters they regulate. We therefore examined the percentage of promoters with multiple hits for specific target sets. All the motifs mentioned above as having tight selection (low σ) appear exactly once in all of the promoters, while motifs with less tight selection are occasionally repeated in promoter regions (Swi6 is repeated in 11% of its promoters, Pho4 and Tye7 in 7%, Msn2 and Mbp1 in 5%, and Msn4 in 5%). While the number of cases is too limited to reach a clear statistical conclusion on the relation between redundancy and selection, it can be hypothesized that for many TFs, redundancy may be high (including multiple hits and possibly also low specificity binding sites), and that such redundancy can alleviate some of the selective pressure on individual loci.

Another possible explanation to low selective pressure on the targets of some critical TFs may be that while some of the physical targets of these TFs are functionally essential and therefore under strong selection, other targets are evolutionarily transient and do not have a major functional role, although they bound specifically in vivo. This hypothesis should be further explored using experimental data on TF binding for additional yeast species.

### Quantifying Selection on Boundary Motifs

**Figure 5:**
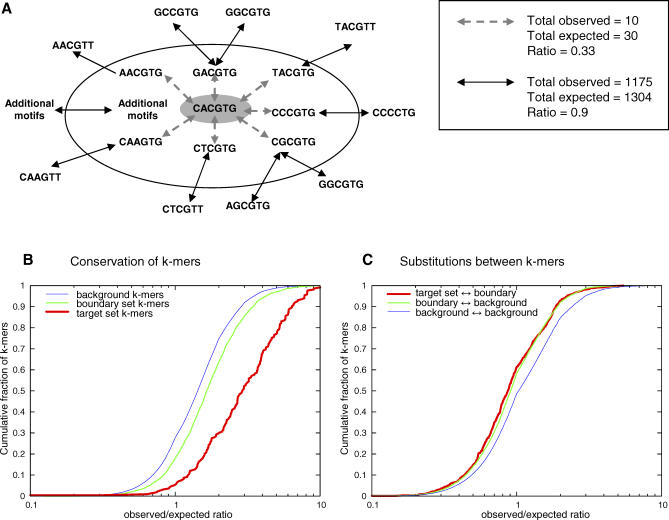
Selection on Target k-mer Sets (A) The selection on single nucleotide substitutions. Our model predicts substitutions between target k-mer set k-mers (inner circle) and boundary k-mers (ring) to be under negative selection, and substitutions between boundary k-mers and background k-mers (outside the ring) or between two background k-mers not to be under selection. The right hand box shows observed and expected counts for the substitutions in one example (CACGTG in the *cerevisiae-mikatae* alignment), as well as the ratio between the observed and expected counts. (B) Ratio of observed and expected conservation. The plot shows the cumulative distribution of the ratios between the observed number of conserved motif appearances and the number expected given a neutral model. Shown are the distributions for three different sets of motifs: target k-mer set (red), boundary (green), and background (blue). A full concordance with the neutral model would have resulted in a perfect lognormal distribution. The background distribution is the closest to lognormal, but still shows bias toward increased conservation due to the clustering of mutations in yeast promoters (unpublished results). Target k-mers are conserved above what is expected. This is evident from looking at the observed–expected ratio distribution, and also when comparing it to the observed–expected ratio distribution for background k-mers (KS (Kolmogorov-Smirnov test) = 0.42 for difference from background k-mers, *p* < 3.8e-43), and the same is true for boundary k-mers, but to a lesser degree (KS = 0.09 for difference from background k-mers, *p* < 3.4e-36). (C) Ratio of observed and expected substitutions. The plot shows the cumulative distribution of the ratios between the observed number of substitutions between motifs and the number expected by a neutral model. Shown are plots for substitutions between target k-mers and boundary k-mers (red), between boundary and background k-mers (green), and between background k-mers (blue). Substitutions between target k-mers and boundary k-mers appear less than expected. Again, this is evident when looking both at the ratio distribution (more than 60% of the data points have ratio < 1) and at its difference from the distribution for substitutions between background k-mers (KS = 0.14, *p* < 2.3e-13). Substitutions between boundary k-mers and background k-mers are also occurring less often than expected, but to a somewhat lesser extent (KS = 0.1, *p* < 3.8e-169). As with the conservation data, the background distribution here is not lognormal, due to the non uniform distribution of mutations.

The evolutionary model we described implies three evolutionary regimes on motifs: k-mers can a) be functional sites (part of a target k-mer set), b) be one substitution away from becoming a functional site (boundary k-mers), or c) be at a distance of two substitutions or more from any target k-mer set, and thus behave in a neutral manner (background k-mers) (Figure 5A). According to our basic assumptions, only substitutions between target k-mers and boundary k-mers are subject to selection. Consequently, we predict functional sites to be highly conserved, and boundary k-mers to be slightly conserved—not due to functionality but due to possible selection against binding site emergence. As discussed above, in cases where our modeling assumptions are too restrictive, we may be classifying as boundaries certain k-mers that are in fact weak binding sites. In these cases, we expect some selection to act on substitutions between such boundary k-mers and background k-mers.

To try to characterize the global effects of selection on boundary k-mers, we compared the degree of conservation of target k-mer set, boundary, and neutral k-mers in the literature-based model. This was done by testing how often motifs from each of these groups appear conserved, compared to what is expected given a neutral model. As shown in Figure 5B, the observed conservation of target k-mers is far above what we expect from a neutral model. A weaker but still significant increase in conservation is observed for boundary k-mers, possibly due to weak selection on binding site appearance, or more likely because of mild selection on weak but functional sites. We next examined the substitutions between target k-mers and boundary k-mers, and between boundary and background k-mers, using again the number of observed substitutions compared to the number expected by a neutral model. As shown in Figure 5C, substitutions between target k-mers and boundary k-mers are occurring much less than expected given the neutral model. We observe a slightly weaker, yet similar pattern for substitutions from boundary to background k-mers. At least some of the boundary k-mers in our model may therefore be functional and under some weak selection, forming together with a target k-mer set a TFBS recognition model that is more complex than our simple assumptions.

### Percentage of Sequence under TFBS Selection

Based on the literature model, 1.77% of the promoter sequence is covered by a TFBS. Using the de novo model, the fraction is 2.36%. These models may be extremely incomplete, but even using the entire repertoire of motifs in the MacIsaac study (without conservation or LLR constraints), the fraction is only 3.24%. It is therefore reasonable to conclude that only a small fraction of the promoter sequences is under tight selection against losing high-specificity binding sites.

Previous global studies on the selection on yeast promoters [19] estimated that about 30% of the sequence in S. cerevisiae is under selection. The gap between these estimates and the scarcity of TFBSs can be explained in several ways. Weak selection may affect low-affinity or weakly functional [20] boundary k-mers, and, in fact, when considering target set k-mers and boundary k-mers together, they cover 27.1% of the sequence in the literature-based model and 29.2% in the de novo model. Another possible factor contributing to the selection, which is not included in our model, are forces determining chromatin structure [18,21].

## Discussion

In this study we introduced a new probabilistic model for the evolution of promoter regions that takes into account the combined effects of multiple TFs. We developed an algorithm for calculating the likelihood of a model given pairwise alignments of promoters of orthologous genes from two species. Additionally, we developed algorithms for learning maximum likelihood model parameters. We applied our algorithms to *Saccharomyces* promoter regions, first inferring a model that summarized previously characterized TF specificities in yeast into one principled evolutionary model. We then applied our methods to learn a full model from scratch. We analyzed the patterns of selection on promoter regions as revealed by these models. Specifically, we used our models to study the intensity of selection on TFBSs and to estimate the amount of promoter region under selection due to high specificity TFBSs.

Given our results, it is evident that even on very short evolutionary time scales transcriptional regulation in yeast is highly dynamic. Indeed, the selection factors we computed for almost all TFBSs are higher (less tight) than what we might expect from functionally essential loci (averaging around 0.5). On average, the calculated selection seems to be weak, even if we restrict the analysis to functionally validated sites (ChIP targets). On the other hand, we observed a significant gap between the amount of selection we can account for using characterized TFBSs and the overall reported selection on yeast promoters. Taken together, it can be hypothesized that much of the functionality of transcriptional networks is encoded in ways other than strong TFBSs, and that due to high levels of redundancy, binding sites are under continuous remodeling [22–25]. Rather than being a deterministic and sparse network, transcriptional programs may be shaped as dense, noisy networks that are continuously changing during evolution.

Much of the past research on comparative methods for noncoding regions has focused on the evolutionary dynamics of TFBSs, as they have relatively well-defined features and a clear functional role. In addition to conservation-based methods for identifying TFBSs [4,5], several studies introduced methods for detecting TFBS motifs using phylogeny-based probabilistic models that distinguish between the evolution of TFBSs and of the neutral background [26,27]. Other studies associated the evolutionary rate with the physical strength of TF–DNA interactions [11,15,16]. These studies strongly motivated the development of a general model for the evolution of regulatory regions in the presence of TFBSs.

The more general approaches for context-aware molecular evolution were so far limited to modeling of neutral evolutionary processes [28–30], or tailored to rigidly structured protein coding regions [31], RNA coding genes [32], or CpG dinucleotides [33]. The model we develop here is a step toward overcoming the major computational difficulties in handling the evolution of large regions with heterogeneous function (many binding sites, sparsely and non-uniformly arranged). To make the model more realistic, additional effects will have to be considered, including binding sites with variable affinities, chromatin structure, combinatorial regulation, and more. Computationally, the adaptation of our methods for computing likelihood and learning models to general phylogenies will require solution of a difficult ancestral inference problem [34]. Analysis of more than two species will allow better understanding of the different dynamics associated with binding site gain and loss (which cannot be distinguished based on pairwise alignments). We hope that further work on these challenges will open the way to faithful modeling of regulatory evolution in higher eukaryotes.

## Methods

### Evolution under the effect of a TF recognition code.

Define a *TF recognition code* to be a collection of sets *C_1_... C_m_* where each C*_t_* is a set of words of length *k_t_*. *C_t_* is called the *target k-mer set* of the *t*-th TF. Typically, *C_t_* will consist of highly similar words. We define an indicator function *β_t_*(*s,i*) whose value is 1 if the *i*-th position in sequence *s* falls inside a word from target k-mer set *C_t_* (i.e., if the substring *s*[*i−j,..,i−j*+*k_t_*-1*)*] ∈ *C_t_* for some 0 ≤ *j* < *k_t_*), and 0 otherwise. Every occurrence of a word from *C_t_* in the promoter sequence *s* is declared a binding site of the *t*-th TF. Our model therefore assumes that TFs recognize all loci-bearing words from their target k-mer sets, and no other loci. It also assumes that all words from the same target k-mer set behave identically, and that the target k-mer sets (and therefore the DNA binding domains of the TFs) remain constant during the evolutionary period considered. See Figure 1A for an illustration of a TF recognition code. A word of length *k_t_* that does not belong to any target k-mer set is called a *boundary k-mer* for TF *t* if it is one substitution away from some k-mer in *C_t_* (see Figure 1B).

Given two aligned sequences *s*_1_ and *s*_2_, we define a model of the evolution of *s*_1_ into *s*_2_, based on the TF recognition code model introduced above. We assume that each nucleotide in the sequence is evolving independently with a neutral substitution rate, with the exception that substitutions that change the regulatory role of a nucleotide—either eliminating or introducing a TFBS—are selected against. We model neutral evolution using a standard instantaneous nucleotide substitution rate matrix *Q_b_*, defining *Q_b_*(*c*_1_ → *c*_2_) as the neutral rate of substitution from nucleotide *c*_1_ to *c*_2_. The effect of selection on TFBSs of the *t*-th TF is formalized using a *selection factor* 0 < σ*_t_* < 1. We assume that a substitution with neutral rate *p* has a reduced rate σ*_t_ p* whenever it adds or removes a binding site for the TF *t*. The instantaneous rate of mutation at the *i*-th position of a sequence *s* is therefore defined by:

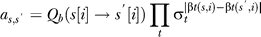
where *s′* is equal to s in all positions but *i*.

To compute the probability of evolving from an entire sequence *s* to a sequence *s′*, we have to combine the effects of multiple TFBSs and of the neutral process, taking into account the epistasis between nucleotides at nearby positions that code for the same TFBS (Figure 1C). The interactions between loci make the common approach of decomposing the sequence into independently evolving loci impossible, since, for example, a mutation in one position that falls within a TFBS may abolish binding and completely alleviate the selective pressure on all other positions. To enable the computation in practice, we will rely on a parsimonious assumption that we outline below. We will show elsewhere how to derive this approximation from an unrestricted Markov model. We also note that our model is defined as symmetric and reversible, so the generalization to phylogenetic trees is direct.

### The parsimonious Markov model.

Given two aligned sequences *s*_1_, *s*_2_, we define the set *S*(*s*_1_,*s*_2_) as the collection of sequences *ŝ* such that for all positions *i* either *ŝ*[*i*]= *s*_1_[*i*] or *ŝ*[*i*]= *s*_2_[*i*]. Our simplifying approximation is that in the evolutionary trajectory between *s*_1_ and *s*_2_, only sequences in *S*(*s*_1_,*s*_2_) have occurred. Those sequences are called *parsimonious with respect to s*_1_, *s*_2_ *.* Given a TF recognition code, we say that two positions *i*,*j* are *epistatic* if there exists a state *s*′ ∈ *S*(*s*_1_,*s*_2_) and a TF *t* such that *s*′[*i*] and *s*′[*j*] are part of the same appearance of a k-mer from *C_t_*, or a boundary k-mer for TF *t*. An *epistatic block* is defined to be a maximum interval in the alignment in which every two adjacent positions are epistatic. The simplest epistatic block is a single neutral nucleotide, which does not interact with any TF in the extant sequences or in any parsimonious trajectory between them. The next basic case is that of an interval including exactly one TFBS (compare Figure 1C). In general, when there are several sites overlapping each other, the epistatic blocks define the smallest possible units for which we can compute the model likelihood independently. It can be shown that under the parsimonious assumption, the probability of *s*_1_ evolving into *s*_2_ equals the product of the probabilities of evolution in each of the epistatic blocks.

Working inside an epistatic block, we still have to compute the probability of evolving from *s*_1_ to *s*_2_ in time *t* using only sequences in *S*(*s*_1_,*s*_2_). This can be done by constructing a continuous time Markov model on all the parsimonious states of the block and an additional state designated OUT which absorbs all probability of transitions to nonparsimonious states. The total probability can then be computed using exponentiation of the model's rate matrix [35]. In practice, we further approximate the matrix exponential using a time-quantized Markov model as follows: Define a time step *dt* as *t*/L where L is larger than the number of point mutations between *s*_1_ and *s*_2_. The background mutation probabilities *P_b_* for the time step *dt* are computed by exponentiation of the background rate matrix *Q_b_*. We define model states *u_t,s_* for times *t* = 0 … *L*−1 and sequences *s* ∈ *S*(*s*_1_, *s*_2_) and add the special state OUT. The transition probabilities *Pr*(*u_t,s_* → *u_t_*_+1,s′_) are defined as

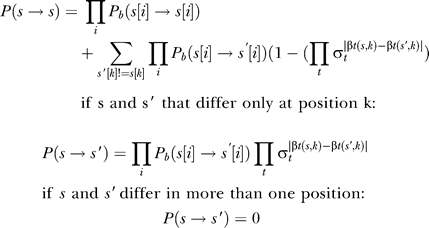



We complete the transition probability from each state to 1 by adding an appropriate transition to the state OUT. Using this model, we can approximate the probability in one epistatic block by standard dynamic programming in the discrete Markov model.

In summary, to compute the likelihood of a model (target k-mer sets and selection factors) given a set of pairwise alignments, we work in phases (see Figure 1D). First we partition the alignment into epistatic blocks by searching for target k-mer set or boundary k-mers in the aligned sequences and their parsimonious combinations. We then compute the log-likelihood of each block using the discrete Markov model, and sum the contributions. Note that in a typical scenario, a substantial fraction of the sequence is neutral with respect to the model, which translates to epistatic blocks of length one. When computing the log-likelihood ratio of some model versus the null model, we can ignore all of these single-nucleotide blocks. Note that to compute log-likelihood ratios, we apply the time-quantized parsimonious approximation to both the null and the target models, thereby avoiding biases introduced by the approximation.

### Model learning.

To learn a maximum likelihood model given a set of pairwise alignments, we devised a multiphase greedy algorithm. Formally, given a set of pairwise alignments and assuming a background neutral substitution model *Q_b_* (which we compute directly from the alignments), we wish to find target k-mer sets *C*_1_ ... *C_m_* and their selection factors σ_1_ ... σ*_m_* such that the model likelihood is optimal. The algorithm optimizes the selection factors given fixed target k-mer sets, and repeatedly attempts to add additional target k-mer sets and to refine existing ones. The key point in the implementation of the algorithm is in careful weighting of candidate motifs for extending the recognition code, since considering all motifs at each step of the algorithm is infeasible. A detailed description of the algorithm is available in Text S1 and Figure S1. Additional details and data on simulation experiments is available in [36].

Additional methods are in described in Text S1.

## Supporting Information

Figure S1 LLR Distribution of the 1st, 3^rd^, and 5th Ranked Motifs in Shuffled Data Experiments (34 KB EPS)

Table S1 Alignment of Data Statistics (25 KB DOC)

Table S2 The De Novo Model (132 KB DOC)

Table S3 Effect of Binding Affinity Threshold on σ and LLR Values for Known Model (136 KB DOC)

Text S1 Supplementary Methods (43 KB DOC)

## Abbreviations

LLR: log-likelihood ratio
TF: transcription
TFBS: transcription factor binding site
