# Supplementary material for: Evolution and Selection in Yeast Promoters: Analyzing the Combined Effect of Diverse Transcription Factor Binding Sites
Source: PLoS Comput Biol. 2008 Jan 11;4(1):e7. doi: 10.1371/journal.pcbi.0040007 (PMC2186363; doi:10.1371/journal.pcbi.0040007)
Supplement: Supplementary file 1 [file pcbi.0040007.sd001.doc]

**Text S1: Supplementary methods**

**Model Learning.**

Given a set of pairwise alignments and assuming a background neutral substitution model P_b_ (which we compute directly from the alignments), we wish to find target k-mer sets {C_1_ ... C_m_} and their selection factors σ_1_ ... σ_m_ such that the model likelihood (approximated as described in the main text) is optimal.

**Parameter Inference.** Assume first that we are given target k-mer sets {C_1_ ... C_m_} and a set of pairwise alignments, and we wish to infer the maximum likelihood parameters σ_1_ ... σ_m_. The problem is difficult to solve analytically, given the different effects each σ_t_ has on each epistatic block (e.g., when there are overlapping binding sites). We therefore work in iterations, focusing each time on a single TF t and fixing σ_i_ for i ≠ t. Optimizing σ for a single TF is still not completely straightforward. We currently use a standard binary-search algorithm to find σ_t_ that gives a maximum likelihood for the model. Empirically, this approach performs well since the optimization function is nearly convex.

**Complete model Learning.** Learning the target k-mer sets from scratch is a more difficult problem, involving a vast solution space and relatively few restrictions on the number of TFs, the size of their target k-mer sets and the relations among them. We approach this problem heuristically, working in iterations and successively adding new target k-mer sets or updating the structure of existing ones. Since the number of possible target k-mer sets is extremely large, and since likelihood calculation and σ optimization are computationally demanding, we cannot search all possible changes per iteration. Instead, we use efficient pre-processing of all candidate k-mers (limiting k to a reasonable range) followed by a greedy search in the model space, guided by the results of the pre-processing.

The learning algorithm is organized as follows:

1) Compute statistics on all k-mers and obtain a small set of promising candidates.

2) Sort candidates by approximating their potential contribution to the model likelihood.

3) Iteratively try to add candidates to the model according to their approximated contribution. In each iteration, a new singleton target k-mer set containing the candidate is added to the model, and then expanded with highly similar k-mers.

An addition to the model is accepted if and only if it increases the model likelihood.

We define n_m_ to be the number of times k-mer m appears in the promoter sequences of the first species. n_m,m'_ is the number of times m appears in the first species aligned against m' in the second species. n^p^_m,m'_ is the number of times we expect m' to appear in the second species aligned against m in the first species, according to the neutral model. It is calculated as: n^p^_m,m'_ = n_m_ * P_b_(m 🡪 m').

In what follows, we shall use P_M_(m 🡪 m') to denote the probability that k-mer m will be aligned against m' according to the model M. In the neutral model b, this probability is computed by multiplying independent substitution probabilities in each position. In a non-trivial model, for certain k-mers the neutral probability is reduced by selection factors.

In the first stage of the algorithm we screen for conserved k-mers (with k ranging over some pre-selected range). Assuming n_m,m_ is a binomial variable with success probability P_b_(m 🡪 m), we compute a p-value for the conservation of each k-mer, conservatively correcting for multiple testing using the Bonferroni approach. k-mers with p-value ≤ 0.005 are considered as candidates in the subsequent stages. Additionally, we filter out candidates with less than a minimal number of hits (10 in this work). We call the set of remaining candidates F. For the yeast data used in this study, using gapless k-mers of size 6-12, F contains a few hundred candidates.

In the second stage of the algorithm, we approximate the likelihood contribution of adding a k-mer to the model by roughly estimating its selection factor σ, and approximating the likelihood of the current model extended with the additional k-mer. We use M to denote the current model, and M' for the extended model.

For a singleton target k-mer set C_t_ = {m}, we set σ^appx^_t_ = ∑_m'≠m_n_m,m'_ / ∑_m'≠m_n^p^_m,m'_. This approximation assumes that in the course of evolution, ∑_m'≠m_n^p^_m,m'_ appearances of m were affected by a substitution, but only ∑_m'≠m_n_m,m'_ of them were fixated. The proportion of fixated mutations is an approximation to the selection factor. In reality, the situation is much more complex due to the effect of multiple substitutions, overlapping binding sites and selection against the emergence of TFBSs. The approximate likelihood ratio of extending the model with k-mer m is then calculated as:

$$\frac{\prod_{m'} P_{M'}\left(m\to m'\right)^{n_{m,m'}}}{\prod_{m'} P_{M}\left(m\to m'\right)^{n_{m,m'}}}$$

The approximated likelihood contribution is computed as if all appearances of m form independent epistatic blocks and ignores the effects of the new model on existing motifs with occurrences overlapping m. Under this assumption, there is a small set of possible m’ values,each possibly appearing multiple times, so the LLR can be quickly calculated once for each m’. After calculating the estimated contribution for each m ∈ F, we sort F according to this score, and use the ordered list in the next stage.

In the third stage, we iteratively try to add and extend target k-mer sets, using the order computed in the previous stage. For each candidate k-mer m, we start by adding m as a new singleton target k-mer set in the model. Then, we perform a local search to add k-mers to the new target k-mer set. First we try to add k-mers that are one substitution away from m. An addition is accepted only if its LLR is positive. Then, we continue to expand with k-mers that are combinations of existing k-mers in the target k-mer set. Following the addition and expansion of a new target k-mer set, we return to Stage 2 and re-calculate the approximated contribution of each candidate.

We continue iterating Stages 2 and 3 until no more candidates are left.

**Data.** We downloaded multiple alignments of *S. cerevisiae* with six other *Saccharomyces* species from the UCSC Genome Browser (version 1, Oct. 2003) (Karolchik, Baertsch, Diekhans, Furey, Hinrichs, Lu, Roskin, Schwartz, Sugnet, Thomas et al. 2003). Using gene annotations, we extracted pairwise alignments of *S. cerevisiae* promoters with each of *S. paradoxus*, *S. mikatae* and *S. bayanus*, using up to 300bp upstream of the Translation Initiation Start.

**Significance of LLR values.** To test the significance of the LLR values of target k-mer sets, we created 1,000 randomly shuffled alignments and applied our learning algorithm to them (**Figure S1**). We then sort the resulting N target k-mer sets by decreasing LLR, and set the LLR for the i-th target set, where i>N, to 0. We can compute a bound on the p-value of an LLR using the empirical distribution of LLR scores of the I’th motif. Note that because the algorithm is filtering candidates motif using their conservation z-scores (phase 1 of the algorithm), we never find more than few target k-mer sets in the shuffled data. Comparing the i-th motif in our de-novo model to the 1,000 i-th motifs resulting from this analysis, we conclude that all the motifs we detect are significant with respect to the null (independence) hypothesis. It is clear, however, that low scoring target k-mer sets in our de-novo model, while significant given the null hypothesis, represent evolutionary effects that are not necessarily related to TFs. For example, the distribution of k-mer conservation is biased by the non uniform distribution of mutations in the yeast promoters. Since our main goal is to establish general characteristics of the evolutionary process and not to find novel TFBS motifs, we prefer more detailed models on more stringent ones. We are not suggesting that these low scoring individual motifs are robust or biologically meaningful by themselves.

**Computing z-scores for separation between target sets.** To directly test the significance of separation between two similar target k-mer sets, we compared the observed number of substitution between k-mers in the two sets to the number of substitution expected given a model that identified both sets as one. According to such a model, the probability of substitutions between any two motifs in the two target sets is simply the neutral probability. We computed a Z score for the difference between the observed and expected inter-sets substitutions assuming the number is binomially distributed with sufficiently many expected success events.

**References**:

Bulyk, M.L., A.M. McGuire, N. Masuda, and G.M. Church. 2004. A Motif Co-Occurrence Approach for Genome-Wide Prediction of Transcription-Factor-Binding Sites in Escherichia coli. *Genome Research* **14:** 201-208.

Harbison, C.T., D.B. Gordon, T.I. Lee, N.J. Rinaldi, K.D. Macisaac, T.W. Danford, N.M. Hannett, J.B. Tagne, D.B. Reynolds, J. Yoo et al. 2004. Transcriptional regulatory code of a eukaryotic genome. *Nature* **431:** 99-104.

Karolchik, D., R. Baertsch, M. Diekhans, T.S. Furey, A. Hinrichs, Y.T. Lu, K.M. Roskin, M. Schwartz, C.W. Sugnet, D.J. Thomas et al. 2003. The UCSC Genome Browser Database. *Nucl. Acids Res* **31:** 51-54.
