# Supplementary material for: Evolution and Selection in Yeast Promoters: Analyzing the Combined Effect of Diverse Transcription Factor Binding Sites
Source: PLoS Comput Biol. 2008 Jan 11;4(1):e7. doi: 10.1371/journal.pcbi.0040007 (PMC2186363; doi:10.1371/journal.pcbi.0040007)
Supplement: Supplementary file 2 [file pcbi.0040007.st001.doc]

**Table S**1: Alignment data statistics

|  | similarity | gaps |
| --- | --- | --- |
| *cerevisiae – paradoxus* | 83.2% | 4.3% |
| *cerevisiae – mikatae* | 74.2% | 7.2% |
| *cerevisiae - bayanus* | 67.1% | 11.2% |
