# Supplementary material for: Evolution and Selection in Yeast Promoters: Analyzing the Combined Effect of Diverse Transcription Factor Binding Sites
Source: PLoS Comput Biol. 2008 Jan 11;4(1):e7. doi: 10.1371/journal.pcbi.0040007 (PMC2186363; doi:10.1371/journal.pcbi.0040007)
Supplement: Supplementary file 3 [file pcbi.0040007.st002.doc]

**Table S2: The *de-novo* model**

**For each target k-mer set in the model, we present: (1) The k-mers in the target k-mer set, (2) a z-score for the number of substitutions inside the target k-mer set, (3) the total number of hits in the data, for k-mers in the target k-mer set and their reverse complement, (4) the  value inferred for the target k-mer set, (5) the log-likelihood ratio between the models before and after the addition of the target k-mer set, and (6) similarity to known motifs.**

| **target k-mer set** | **z-score** | **hits** | **σ** | **LLR** | **known motif match** |
| --- | --- | --- | --- | --- | --- |
| AACGCG ; GACGCG ; TACGCG ; ACCGCG ; ATCGCG ; AACCCG ; AACGGG ; ACCGGG ; ACCCGG ; ACCCCG ; GCCGGG ; GCCGCG ; TACGGG ; TACCCG ; TCCGGG ; TCCGCG ; TTCGGG ; TTCGCG ; TTCCGG | 38.72 | 2937 | 0.63 | 498.69 | SCB/MBP1 |
| TTACCCG | 27.90 | 248 | 0.10 | 372.44 | REB1 |
| GATGAG ; GATGAC | 22.23 | 984 | 0.51 | 277.40 | PAC |
| TATATAA ; TATAAAA ; TATATAC | 20.95 | 1950 | 0.72 | 205.91 | TBP |
| TGAAAAA ; TGAAAAT | 16.00 | 1553 | 0.69 | 145.73 |  |
| CCCCTT | 11.70 | 317 | 0.48 | 114.44 | STRE MSN2/4 |
| CACGTG | 17.77 | 204 | 0.27 | 89.15 | MET28 TYE7 PHO4 CBF1 |
| TAGGGG ; CAGGGG ; TAAGGG | 16.02 | 605 | 0.62 | 77.81 | MSN2 STRE |
| TAGCCGCC ; TAACCGCC | 12.52 | 50 | 0.12 | 70.02 | UME6 |
| TTACCCT ; TTACCCC | 12.59 | 193 | 0.46 | 67.60 | REB1 |
| TTTCTTTTT ; TTTATTTTT | 9.98 | 546 | 0.68 | 62.65 |  |
| TTTGCCACC | 13.66 | 36 | 0.12 | 59.82 | RPN4 |
| TGACTC ; TGACAC | 9.46 | 537 | 0.67 | 59.58 | GCN4 |
| TTGCCGC ; TTGCCAC; TTTCCGC ; TCGCCGC ; TCGCCAC | 13.03 | 406 | 0.61 | 58.42 | RPN4 |
| TCGTTTA | 12.66 | 156 | 0.50 | 52.12 | SRE |
| TATTTATA | 11.72 | 222 | 0.56 | 48.78 | RLM1 |
| CGATGGG ; CGATGAG | 20.80 | 268 | 0.50 | 40.53 | PAC |
| GATAAG | 10.27 | 462 | 0.68 | 39.79 | GZF3 GLN3 GAT1 |
| GAAATTTTT | 10.63 | 101 | 0.45 | 37.00 | RRPE |
| GTTTGTTT ; CTTTGTTT ; TTTTGTTT | 9.91 | 584 | 0.77 | 31.45 |  |
| GGCATCG ; CGCATCG | 8.97 | 101 | 0.51 | 28.28 | INO2/4 |
| TGTGGC ; GGTGGC ; TGCGGC ; TGTCGC ; GGCGGC ; GGCCGC ; TGCCGC | 15.05 | 1126 | 0.83 | 28.19 |  |
| GCGATGAGATG ; GCGATGAGATA | 16.22 | 27 | 0.06 | 27.62 | PAC |
| GTAAACAA ; GTAAACAG | 8.09 | 136 | 0.58 | 24.88 | SFF FKH1/2 |
| ACACAAAA ; CCACAAAA | 9.91 | 113 | 0.57 | 23.59 | MSE |
| GTGTGG ; GTGAGG ; GTGGGG | 8.53 | 500 | 0.78 | 23.36 |  |
| GGACCCT ; AGACCCT | 7.68 | 107 | 0.54 | 23.25 | NRG1 |
| CCGAGA ; CCGACA ; CCGAGG ; CCGAGC ; CCGACG | 9.54 | 550 | 0.76 | 23.20 |  |
| ACAATAG | 6.30 | 250 | 0.70 | 23.06 | MAT ROX1 |
| TTTGTGTC | 8.52 | 45 | 0.39 | 23.00 | MATα MCM1 MIG1 TUP1 |
| GCGATGG | 5.87 | 67 | 0.50 | 19.81 | PAC |
| AAATCCG ; ACATCCG ; AACTCCG | 9.34 | 178 | 0.68 | 18.14 |  |
| AGAGATGAG ; TGAGATGAG | 17.17 | 94 | 0.50 | 15.78 | PAC |
| TGGGTGC ; GGGGTGC ; TGGTTGC | 6.14 | 116 | 0.65 | 15.67 | AFT2 |
| TATTTACA | 7.81 | 110 | 0.61 | 15.39 | RLM1 |
| GTGGGGTA | 9.42 | 11 | 0.10 | 15.35 | TUP1 |
| TTTGTGAC | 6.36 | 38 | 0.46 | 13.65 | MATα MCM1 MIG1 TUP1 |
| GAAATTTCA ; CAAATTTCA ; GATATTTCA ; GAAATCTCA ; GAAATTCCA ; GATATCTCA ; CATATTCCA | 8.70 | 126 | 0.69 | 12.95 | HSTF |
| CCGATA | 6.82 | 165 | 0.67 | 12.72 | HAP1 |
| CGCAAAA; CACAAAA | 8.03 | 359 | 0.79 | 11.28 | MSE |
| GGCGCTA | 6.61 | 48 | 0.53 | 11.01 | SNT2 |
| CACGCAA ; CACGTAA ; CACGCAG | 6.37 | 156 | 0.74 | 9.95 |  |
| TTTCGTGT | 9.15 | 50 | 0.57 | 9.65 | SWI4 SCB |
| CGCGGGG | 9.33 | 24 | 0.36 | 8.25 | SUT1 |
| AAGGGAG | 6.07 | 103 | 0.71 | 8.24 |  |
| CGGAAAAA ; CGGAGAAA | 7.32 | 116 | 0.71 | 7.83 | RGT1 ECB |
| AAAGTGAAAAA | 7.47 | 37 | 0.54 | 7.83 |  |
| TCGGCC ; TCGGGC ; TCGGCA | 8.59 | 397 | 0.81 | 7.80 | RDS1 |
| AAATAGAG ; AAATAGGG | 5.53 | 118 | 0.74 | 7.76 |  |
| ATGACT ; ATGGCT | 7.15 | 728 | 0.88 | 7.73 | ARG81 |
| GCGCGGG | 8.75 | 27 | 0.49 | 7.58 | PDR3 |
| AAAAGAAAC ; AAAACAAAC | 9.70 | 137 | 0.77 | 7.51 | STE11 |
| GCCACCG | 9.29 | 42 | 0.57 | 7.47 | RPN4 |
| AAGATCAA | 6.19 | 73 | 0.72 | 7.27 |  |
| TTCTAGAA | 6.45 | 80 | 0.62 | 6.81 | HSTF HSF1 |
| CCCCAA ; GCCCAA | 6.43 | 354 | 0.84 | 6.50 |  |
| TTTCGCGA | 6.51 | 43 | 0.49 | 6.35 | SWI4 |
| TTCTTCGA ; TTCTTCGG | 5.66 | 100 | 0.75 | 6.21 | STE11 |
| TCAACAA | 5.95 | 235 | 0.83 | 5.25 | SFF |
| AAAGGGTT ; AAAGGGTA | 6.90 | 105 | 0.77 | 5.09 | MSN2/4 |
| TGAAACA | 6.67 | 211 | 0.82 | 4.49 | STE12 |
| CAGCAGC ; CAGCATC | 5.17 | 161 | 0.84 | 4.18 | ACE2 SWI5 |
