# Supplementary material for: Evolution and Selection in Yeast Promoters: Analyzing the Combined Effect of Diverse Transcription Factor Binding Sites
Source: PLoS Comput Biol. 2008 Jan 11;4(1):e7. doi: 10.1371/journal.pcbi.0040007 (PMC2186363; doi:10.1371/journal.pcbi.0040007)
Supplement: Supplementary file 4 [file pcbi.0040007.st003.doc]

**Table S3**: **Effect of binding affinity threshold on σ and LLR values for known**

**model.** We evaluated the known model on three datasets of S. cerevisiae – S. mikatae alignments, obtained by taking only those promoters that passed the binding affinity p-value thresholds of 0.05, 0.01 and 0.001, respectively, according to (Bulyk, McGuire, Masuda and Church 2004; Harbison, Gordon, Lee, Rinaldi, Macisaac, Danford, Hannett, Tagne, Reynolds, Yoo et al. 2004). For each target k-mer set in the known model, we present the LLR and  values computed on each of the datasets.

| **TF** | **consensus** | **0.05 sigma** | **0.05 LLR** | **0.01 sigma** | **0.01 LLR** | **0.001 sigma** | **0.001 LLR** |
| --- | --- | --- | --- | --- | --- | --- | --- |
| ACE2 | ARCCMGCM | 0.92 | 0.05 | 0.74 | 0.31 | 0.37 | 1.54 |
| AFT2 | KGCACCC | 0.28 | 13.77 | 0.20 | 10.65 | 0.13 | 10.28 |
| ARG81 | .TGACTCY | 0.60 | 2.33 | 0.23 | 4.13 | 0.38 | 0.71 |
| ASH1 | CCR.RTCRGG | 0.61 | 0.62 | 0.81 | 0.08 | 0.73 | 0.12 |
| BAS1 | TGACTCY... | 0.29 | 17.73 | 0.26 | 13.67 | 0.14 | 13.92 |
| CAD1 | GCTKACTAAT | 0.23 | 2.04 | 0.54 | 0.21 | 0.62 | 0.12 |
| CBF1 | GTCACGTG | 0.34 | 14.23 | 0.31 | 16.19 | 0.33 | 13.09 |
| CHA4 | TGCGATGAR | 0.30 | 9.94 | 0.43 | 2.10 | 0.30 | 1.17 |
| CIN5 | MTTACRTAA | 0.33 | 11.88 | 0.30 | 10.48 | 0.33 | 2.14 |
| CST6 | TGCATTT. | 0.81 | 0.54 | 0.53 | 1.84 | 0.32 | 1.04 |
| DAL80 | GATAA | 0.80 | 11.60 | 0.82 | 2.88 | 0.77 | 1.84 |
| DAL82 | AAA.WTGYG. | 0.79 | 1.49 | 0.74 | 1.24 | 0.40 | 3.19 |
| FHL1 | RTGTACGGRT | 0.47 | 5.66 | 0.41 | 6.85 | 0.36 | 8.10 |
| FKH1 | ....GTAAACAA | 0.25 | 19.65 | 0.21 | 17.26 | 0.08 | 21.32 |
| GAT1 | AGATAAG | 0.45 | 21.09 | 0.33 | 15.57 | 0.39 | 3.91 |
| GCN4 | RTGACTCA | 0.32 | 22.46 | 0.28 | 22.10 | 0.25 | 21.11 |
| GLN3 | MGATAAGRTA | 0.19 | 8.94 | 0.21 | 4.83 | 0.17 | 3.47 |
| GZF3 | GATAAG | 0.57 | 19.26 | 0.42 | 11.18 | 0.63 | 1.06 |
| HAP1 | CGATAA..SC | 0.09 | 8.48 | 0.10 | 7.66 | 0.12 | 5.49 |
| HAP2 | CCAAT.A. | 0.69 | 4.47 | 0.52 | 6.21 | 0.12 | 10.99 |
| HAP3 | CCAAT | 0.89 | 1.09 | 0.79 | 1.17 | 0.50 | 2.57 |
| HAP5 | CCAAT | 0.90 | 1.37 | 0.85 | 1.27 | 0.72 | 1.04 |
| LEU3 | CCGGTMCCGG | 0.12 | 5.89 | 0.12 | 5.92 | 0.11 | 0.00 |
| MBP1 | ACGCGTC | 0.34 | 18.19 | 0.27 | 18.79 | 0.32 | 12.61 |
| MCM1 | TKTCC..WTT.GGAAA | 0.47 | 1.34 | 0.42 | 1.25 | 0.41 | 1.32 |
| MET31 | .GTGTGK. | 0.97 | 0.04 | 0.77 | 0.71 | 0.94 | 0.02 |
| MSN2 | MAGGGG. | 0.47 | 48.45 | 0.38 | 24.47 | 0.33 | 12.52 |
| MSN4 | AAGGGG. | 0.39 | 37.74 | 0.41 | 13.46 | 0.25 | 8.89 |
| NDD1 | RAARGGSAAA | 0.74 | 0.46 | 0.56 | 1.08 | 0.27 | 1.50 |
| NRG1 | GGACCCT | 0.32 | 13.94 | 0.28 | 9.99 | 0.38 | 4.22 |
| PDR1 | CCGCCGRAWRA | 0.68 | 0.07 | 0.69 | 0.07 | 0.76 | 0.03 |
| PHD1 | RGGCAM | 0.75 | 6.87 | 0.70 | 3.50 | 0.68 | 1.91 |
| PHO4 | CACGTG. | 0.55 | 16.61 | 0.50 | 12.49 | 0.36 | 9.91 |
| RAP1 | ..ACCCR.ACMY | 0.29 | 19.32 | 0.27 | 17.09 | 0.42 | 2.81 |
| RCS1 | RGGTGCA | 0.37 | 16.88 | 0.22 | 15.81 | 0.16 | 15.34 |
| RDS1 | CGGCCG | 0.47 | 3.55 | 0.35 | 2.69 | 0.28 | 2.97 |
| REB1 | TTACCCGS | 0.20 | 76.36 | 0.21 | 59.49 | 0.18 | 43.71 |
| RME1 | Y..AAAGGAA | 0.92 | 0.05 | 0.86 | 0.07 | 0.57 | 0.41 |
| RPH1 | CCCCTTAAGG | 0.61 | 0.26 | 0.58 | 0.17 | 0.89 | 0.01 |
| RTG3 | RTGACKC. | 0.52 | 17.29 | 0.59 | 4.54 | 0.64 | 1.28 |
| SFP1 | RTGTAYGGRT | 0.42 | 11.27 | 0.47 | 5.59 | 0.57 | 1.40 |
| SKN7 | GGSCYRGM | 0.80 | 0.96 | 0.59 | 2.30 | 0.54 | 1.69 |
| SOK2 | .CAGGMAM | 0.82 | 1.22 | 0.70 | 1.39 | 0.81 | 0.08 |
| STB1 | CGCGAAAA | 0.20 | 10.38 | 0.16 | 7.62 | 0.10 | 7.49 |
| STB2 | CGSGTAA. | 0.33 | 10.00 | 0.22 | 6.91 | 0.32 | 2.17 |
| STE12 | TGAAACA | 0.39 | 18.38 | 0.37 | 17.38 | 0.26 | 18.14 |
| STP1 | YGCGGCK. | 0.62 | 2.30 | 0.49 | 1.70 | 0.45 | 1.40 |
| SUT1 | .GCSGGG | 0.57 | 4.17 | 0.46 | 2.12 | 0.55 | 0.95 |
| SWI4 | .CRCGAAA. | 0.31 | 15.71 | 0.25 | 16.01 | 0.11 | 19.28 |
| SWI5 | TGCTGG.. | 0.78 | 0.50 | 0.63 | 1.01 | 0.79 | 0.21 |
| SWI6 | CGCGTY | 0.28 | 31.55 | 0.26 | 22.30 | 0.22 | 22.25 |
| TEC1 | .RCATTCY | 0.80 | 2.02 | 0.61 | 2.91 | 0.42 | 2.77 |
| THI2 | .GMAACYSWWAGARCY | 0.14 | 4.92 | 0.13 | 5.52 | 0.12 | 5.83 |
| TYE7 | .TCACGTG | 0.23 | 15.83 | 0.07 | 15.80 | 0.10 | 10.89 |
| UME6 | TAGCCGCCSA | 0.05 | 16.27 | 0.09 | 9.18 | 0.08 | 9.40 |
| XBP1 | CTCGAGG. | 0.39 | 11.11 | 0.36 | 3.96 | 0.92 | 0.00 |
| YAP6 | TTACRTAA | 0.45 | 6.59 | 0.57 | 1.77 | 0.59 | 0.74 |
| YAP7 | .MTKACTAAT | 0.44 | 8.24 | 0.38 | 7.24 | 0.27 | 9.59 |
| YOX1 | MATTASGTTTCCYRAWMGGG | 0.42 | 0.52 | 0.41 | 0.53 | 0.38 | 0.64 |
| YRR1 | TTTTGTTACSCR | 0.57 | 0.19 | 0.53 | 0.24 | 0.57 | 0.19 |
